# DBU coupled ionic liquid-catalyzed efficient synthesis of quinazolinones from CO_2_ and 2-aminobenzonitriles under mild conditions

**DOI:** 10.1039/d0ra00194e

**Published:** 2020-03-25

**Authors:** Xiang Gao, Jiao Liu, Zhaopeng Liu, Lei Zhang, Xin Zuo, Leyuan Chen, Xue Bai, Qingyun Bai, Xinlin Wang, Anning Zhou

**Affiliations:** College of Geology and Environment, Xi'an University of Science and Technology Xi'an 710054 China gaoxiang@iccas.ac.cn; School of Chemical Engineering and Technology, China University of Mining and Technology Xuzhou 221000 China; A School of Chemistry and Chemical Engineering, Xi'an University of Science and Technology Xi'an 710054 China psu564@139.com

## Abstract

Efficient and green strategy for the chemical conversion and fixation of CO_2_ is an attractive topic. In this work, we reported an efficient catalytic system of organic base coupled ionic liquids that could catalyse the synthesis of quinazolinones *via* cyclization of 2-aminobenzonitriles with CO_2_ under mild conditions (*e.g.*, 60 °C, 0.1 MPa). It was found that 1,8-diazabicyclo[5.4.0]undec-7-ene coupled 1-butyl-3-methylimidazole acetate ionic liquids (DBU/[Bmim][OAc]) displayed excellent performance in catalysing the reactions of CO_2_ with 2-aminobenzonitriles, and a series of quinazolinones were obtained in high yields at atmospheric pressure. Moreover, the ILs had high stability and reusability, and can be reused at least five times without considerable decrease in catalytic activity. This protocol could also be conducted on a gram scale, and may have promising and practical applications in the production of quinazolinones.

## Abstract 2

Efficient and green strategy for the chemical conversion and fixation of CO_2_ is an attractive topic.

## Graphical abstract

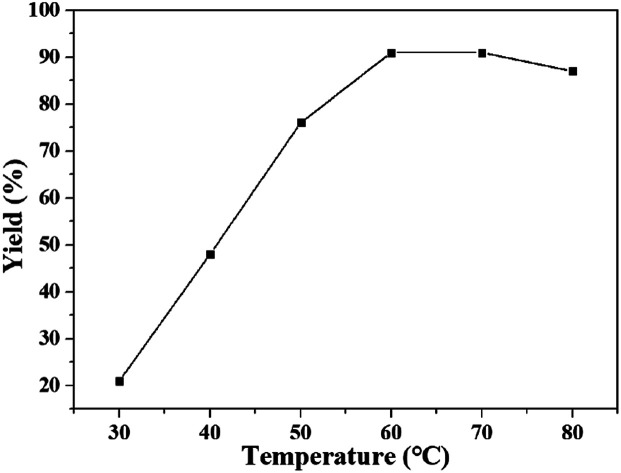

## Introduction

In recent years, the pollution situation for the global natural environment has been increasingly serious and the concept of sustainable development has been mentioned as an important issue.^1^ So, the conversion from pollutants or wastes into valuable chemicals has attracted extensive attention.^2^ As the main greenhouse gas, CO_2_ emission reduction is important for the global climate change and the sustainable development of society. CO_2_ is also a C1 resource with the advantages of being non-toxic, abundant, cheap, easily-available and renewable. This means that while CO_2_ is reduced as much as possible, it can also be converted into energy, materials and chemical products as a carbon resource. In recent years, many value-added chemicals have been synthesized using CO_2_ as a raw material, such as formic acid,^3^ acrylic acid,^4^ benzothiazolone,^5^ dimethyl carbonate,^6^ and others.^7^ Although much progress has been made, the chemical conversion of CO_2_ especially under mild conditions is still a challenge because of its inherent thermodynamic stability and kinetic inertness.

Being a biologically active additive, quinazolinones are an important class of pharmaceutical intermediates and have been widely used in biological and pharmaceutical industries. Various methods for the synthesis of quinazolinones have been reported, involving anthranilic acid derivatives with urea,^8^ anthranilic acid with isocyanate^9^ or potassium cyanate,^10^ 2-nitrobenzamides and triphosgene,^11^ 2-nitrobenzaldiformamide with CO,^12^ and so on. However, these paths inevitably required the use of special or toxic reagents such as phosgene and cyanate. Notably, in recent years, quinazolinones can also be synthesized by the reaction of 2-aminobenzonitriles and CO_2_, which not only effectively uses CO_2_ as a raw material, but also involved more environmentally friendly processes to avoid the use of toxic carbon resources. At present, a number of catalysts have been developed for the synthesis of quinazolinones from 2-aminobenzonitriles and CO_2_, such as TMG (1,1,3,3-tetramethylguanidine),^13^ DBU (1,8-diazabicyclo[5.4.0]undec-7-ene),^14^ MTHP (*N*-methyltetrahydropyrimidine),^15^ TBA_2_[WO_4_] (TBA = tetra-*n*-butylammonium),^16^ choline hydroxide,^17^ MgO/ZrO_2_,^18^ Cs_2_CO_3_,^19^ and so on. Particularly, in 2013, Ma and co-workers^20^ reported that this kind of reaction could proceed smoothly in water medium without any catalysts at higher pressure and temperature (*e.g.*, 160 °C, 14 MPa). However, these above reaction systems have more or less restrictive factors, including harsh reaction temperatures and carbon dioxide pressure. Therefore, the exploration of efficient catalytic systems for the synthesis of quinazolinones from 2-aminobenzonitriles and CO_2_ under mild reaction conditions is highly desirable.

Ionic liquids (ILs) have been widely used in chemical reactions, material synthesis and other fields due to their unique advantages such as good solubility, high thermal stability, low vapor pressure, and easy separation and recovery.^21^ In recent years, a variety of ILs have been explored for catalysing the reaction of CO_2_ with 2-aminobenzonitrile to synthesize quinazolinones, such as [Bmim][OH] (1-butyl-3-methylimidazolium hydroxide),^22^ [Hmim][OH] (1-hexyl-3-methyl imidazolium hydroxide),^23^ [Bmim][OAc] (1-butyl-3-methylimidazolium acetate),^24^ [HDBU^+^][TFE^−^] (1,8-diazabicyclo[5.4.0]undec-7-ene (DBU) as a super base and trifluoroethanol (TFE) as a proton donor),^25^ [HTMG][Im] (1,1,3,3-tetramethylguanidinium imidazolide),^26^ and so on.^27^ Using simple ILs as both the catalyst and solvent for the synthesis of quinazolinones from 2-aminobenzonitriles and CO_2_ could generally make the reaction under mild conditions (0.1 MPa), however, super-stoichiometric amounts of ILs (6 equiv.^25^ or 4 equiv.^26^) was needed. From the view of practical application, these methods were all incompatible with green-chemistry principle, which may reduce their practicality.

**Scheme 1:**
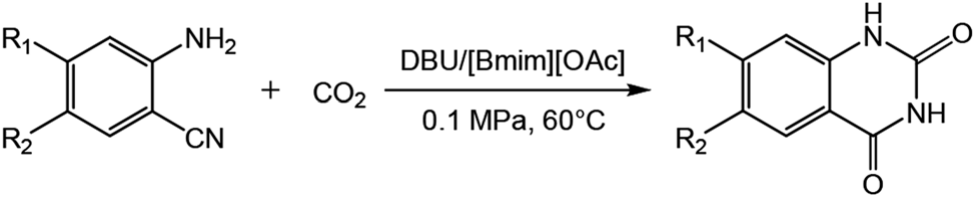
Reaction of CO_2_ and 2-aminobenzonitriles to quinazolinones.

In this work, we have found that the binary catalytic system generated by the coupling of DBU and ILs could effectively promote this kind of reactions using small amount of catalysts under mild conditions (*e.g.*, 60 °C, 0.1 MPa). We studied the reaction *via* different binary catalytic systems of base/ILs. The results showed that the catalytic system of DBU/[Bmim][OAc] could efficiently catalyse the synthesis of quinazolinones from 2-aminobenzonitriles and CO_2_ (Scheme 1), and a series of quinazolinones could be obtained in good to excellent yields at atmospheric pressure. Moreover, the separation of the products from the reaction system was very simple, and the ILs could be reused at least five times without significant loss in catalytic activity. This protocol could also been conducted on a gram scale for the practical synthesis of quinazolinones.

## Results and discussion

The reaction of 2-aminobenzonitrile and CO_2_ was carried out at 60 °C and 0.1 MPa, and the results were shown in Table 1. This reaction did not proceed without catalyst (Table 1, entry 1). Fortunately, a yield of 61% product could be obtained in the presence of single catalyst DBU (Table 1, entry 2). Furthermore, the catalytic activity of the system of base/ILs was studied. Firstly, the combination of DBU and 1-butyl-3-methylimidazolium-based ILs with different anions were investigated for this reaction. Surprisingly, their combination were effective for catalysing this reaction, and the yields of the target product in [Bmim][CF_3_Ac] (1-butyl-3-methylimidazolium trifluoroacetate), [Bmim][NO_3_] (1-butyl-3-methylimidazolium nitrate), [Bmim][ClO_4_] (1-butyl-3-methylimidazolium perchlorate), [Bmim][NTF_2_] (1-butyl-3-methylimidazolium trifluoromethanesulfonyl), [Bmim][BF_4_] (1-butyl-3-methylimidazolium tetrafluoroborate), [Bmim][Cl] (1-butyl-3-methylimidazolium chloride), [Bmim][Br] (1-butyl-3-methylimidazolium bromide), [Bmim][I] (1-butyl-3-methylimidazolium iodine), [Bmim][OAc] (1-butyl-3-methylimidazolium acetate) were 38%, 52%, 58%, 62%, 77%, 68%, 72%, 73% and 91%, respectively (Table 1, entries 3–11). Especially, [Bmim][OAc] showed the best catalytic activity (Table 1, entry 11). This finding indicated that the acetate-based ILs were more effective in this reaction, which maybe due to acetic acid is a weak acid and therefore the acetate-based ILs are alkaline, and the reaction can be promoted by basic catalyst. In addition, the effects of the system of DBU and acetate-based ILs with different cations on this reaction were explored. As seen from Table 1, [Emim][OAc] (1-ethyl-3-methylimidazolium acetate) and [Omim][OAc] (1-octyl-3-methylimidazolium acetate) could catalyse the reaction under experimental conditions, affording quinazolinone in a yield of 79% and 85% (Table 1, entries 12 and 13). For the ILs with Ac- anion, the decrease in the catalytic efficiency showed the following trend: [Bmim][OAc] > [Omim][OAc] > [Emim][OAc], which was probably related to the different cations of ILs. On one hand, as catalyst, the different cations of ILs had different steric hindrance and induced the different ability to form hydrogen bonds with the substrate. On the other hand, as solvent, the different cations of ILs had different viscosity and solubility. Therefore, due to the combined effect of steric hindrance, viscosity and solubility, the DBU coupled ILs system showed different catalytic efficiency. Hence, the catalytic performance of ILs depend on both their cations and anions, and particularly, the anion of ILs has a more significant effect on the reaction. As 0.5 equiv. of ionic liquid was used, the yield of quinazolinone was still 89% (Table 1, entry 14), indicating the great efficiency of the catalytic system of DBU/[Bmim][OAc].

**Table 1:** Reaction of 2-aminobenzonitrile with CO_2_ catalyzed by various DBU/ILs^a^

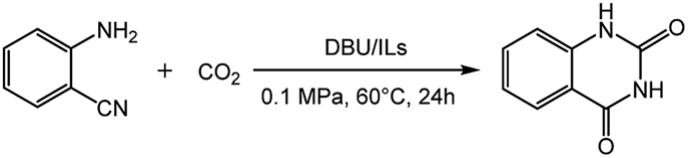		
Entry	Catalyst	Yield^b^ (%)
1	—	0
2	DBU	61
3	DBU/[Bmim][CF_3_Ac]	38
4	DBU/[Bmim][NO_3_]	52
5	DBU/[Bmim][ClO_4_]	58
6	DBU/[Bmim][NTF_2_]	62
7	DBU/[Bmim][BF_4_]	77
8	DBU/[Bmim][Cl]	68
9	DBU/[Bmim][Br]	72
10	DBU/[Bmim][I]	76
11	DBU/[Bmim][OAc]	91
12	DBU/[Emim][OAc]	79
13	DBU/[Omim][OAc]	85
14^c^	DBU/[Bmim][OAc]	89

a Reaction conditions: 2-aminobenzonitrile 2 mmol, DBU 10 mol%, ILs 100 mol%, CO_2_ 0.1 MPa, 24 h, 60 °C.

b Isolated yield.

c [Bmim][OAc] 50 mol%.

The influence of the bases on the yield of quinazolinone was investigated in the catalytic systems coupled of various bases and [Bmim][OAc] at 60 °C and 0.1 MPa, and the results were summarized in Table 2. To our delight, not only inorganic bases like NaOH, KOH, Na_2_CO_3_, Cs_2_CO_3_, but also organic bases like TMG, Imidazole, DBU, DBN which coupled with [Bmim][OAc] could catalyse this reaction, affording quinazolinone in a yield of >78% (Table 2, entries 1–8). Notably, the catalytic system of DBU/[Bmim][OAc] revealed the best catalytic activity, obtaining a yield of 91% quinazolinone (Table 2, entry 7). Using DBN as the base under identical conditions afforded the product with a similar yield as DBU (89%, Table 2, entry 8), possibly due to their similar molecular structure and basicity. Moreover, while decreasing the amount of DBU to 0.05 equiv., the product yield was still high enough of 86% (Table 2, entry 9), further indicating the great efficiency of the catalytic system of DBU/[Bmim][OAc]. Therefore, DBU/[Bmim][OAc] was determined as the best catalysts for the synthesis of quinazolinone.

**Table 2:** Reaction of 2-aminobenzonitrile with CO_2_ catalyzed by various bases/[Bmim][OAc]^a^

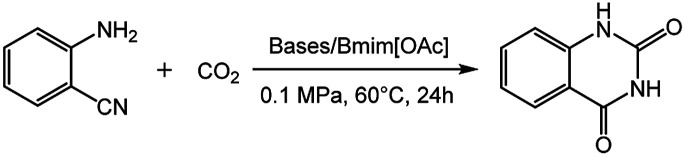		
Entry	Catalyst	Yield^b^ (%)
1	NaOH/[Bmim][OAc]	83
2	KOH/[Bmim][OAc]	83
3	Na_2_CO_3_/[Bmim][OAc]	86
4	CsCO_3_/[Bmim][OAc]	78
5	TMG/[Bmim][OAc]	78
6	Imidazole/[Bmim][OAc]	81
7	DBU/[Bmim][OAc]	91
8	DBN/[Bmim][OAc]	89
9^c^	DBU/[Bmim][OAc]	86

a Reaction conditions: 2-aminobenzonitrile 2 mmol, bases 10 mol%, [Bmim][OAc] 100 mol%, CO_2_ 0.1 MPa, 24 h, 60 °C.

b Isolated yield.

c DBU 5 mol%.

As listed in Table 3, the effects of various solvents on the reaction of 2-aminobenzonitrile with CO_2_ were examined. We observed that the solvents such as THF (tetrahydrofuran), acetonitrile were less efficient under present reaction condition (39% and 65%, Table 3, entries 1 and 2). Likewise, the yields of quinazolinone in solvents of DMSO and DMF were 80% (Table 3, entries 3 and 4). These phenomena indicated that the solvent had a negative influence on the reaction. However, the reaction gave 91% yield of target production under solvent-free condition, demonstrating that solvents would destroy the basicity of the catalytic system and reduced catalytic performance (Table 3, entry 5). Therefore, it could be concluded that the ILs could act as both catalyst and solvent for this reaction system and did not require the participation of other solvents.

**Table 3:** Reaction of 2-aminobenzonitrile with CO_2_ in various solvents^a^

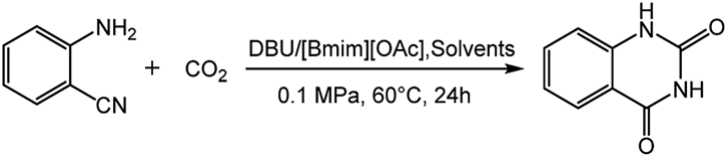		
Entry	Solvent	Yield^b^ (%)
1	THF	39
2	Acetonitrile	65
3	DMSO	80
4	DMF	80
5	None	91

a Reaction conditions: 2-aminobenzonitrile 2 mmol, DBU 10 mol%, [Bmim][OAc] 100 mol%, solvents 2 mL, CO_2_ 0.1 MPa, 24 h, 60 °C.

b Isolated yield.

**Fig. 1:**
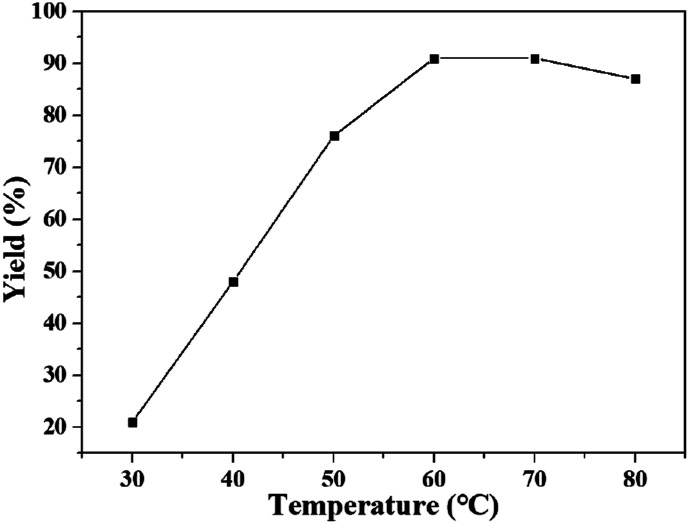
The dependence of the quinazolinone yield on temperature. Reaction conditions: 2-aminobenzonitrile 2 mmol, DBU 10 mol%, [Bmim][OAc] 100 mol%, CO_2_ 0.1 MPa, 24 h. Isolated yield.

The effect of temperature on the yield of quinazolinone was studied in DBU/[Bmim][OAc] at 0.1 MPa for 24 h, and the results were shown in Fig. 1. The reaction was proceed at different temperatures ranging from 30 to 80 °C. It was found that the yield of product increased from 21% to 91% with the increasing temperature from 30 to 60 °C, and did not change when the temperature was increased to 70 °C. However, the yield dropped to 87% when temperature was further increased to 80 °C. This phenomenon maybe because a DBU–CO_2_ complex was formed from carbon dioxide and DBU at normal temperature, which was considered to be an active species of CO_2_ for carboxylation. This DBU–CO_2_ complex was readily thermally decomposed into DBU and carbon dioxide at higher temperature,^14*a*^ inducing a lower yield of the product. Thus, the temperature of 60 °C was considered to be the optimized temperature for the reaction.

**Fig. 2:**
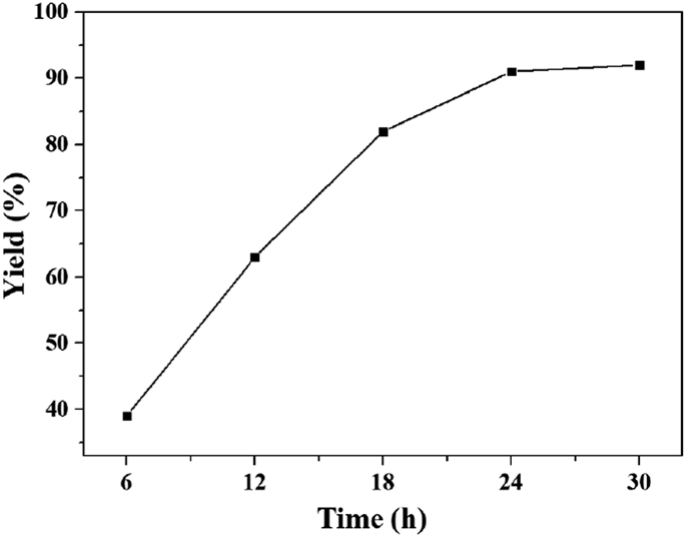
The dependence of the quinazolinone yield on reaction time. Reaction conditions: 2-aminobenzonitrile 2 mmol, DBU 10 mol%, [Bmim][OAc] 100 mol%, CO_2_ 0.1 MPa, 60 °C. Isolated yield.

The influence of reaction time on the yield of quinazolinone at 60 °C and 0.1 MPa was presented in Fig. 2. It was found that the yield of desirable product increased gradually with increasing reaction time. Then the yield of 91% could be obtained in 24 h, and kept unchanged with further prolonging the reaction time to 36 h, indicating that all the reactant could be converted in 24 h. Hence, the reaction time of 24 h was optimal for the synthesis of quinazolinone at 60 °C.

To broaden the potential and general applicability of the proposed approach, the synthesis of quinazolinones from CO_2_ and a series of different 2-aminobenzonitrile derivatives were examined under the optimal reaction conditions, and the results were shown in Table 4. It was observed that the various 2-aminobenzonitrile derivatives with different electron-donating and electron-withdrawing groups could been converted into the corresponding quinazolinones in good to high yields. The reaction of 2-aminobenzonitrile with CO_2_ provided 91% yield of quinazolinone under the selected reaction conditions. The substrate with the electron-donating group such as –OMe afforded 7-methoxyquinazoline-2,4(1*H*,3*H*)-dione with a higher yield of 95%, suggesting that the electron-donating groups improved the activity of 2-aminobenzonitriles while reacted with CO_2_ (Table 4, entry 2). Meanwhile, it was found that most of all the halogen substituents such as –Cl and –Br efficiently converted into corresponding quinazolinones and the yields were 98%, 90% and 88%, respectively (Table 4, entries 3–5). The above finding indicated that the substituents in the phenyl ring of diamines considerably influenced their activities to react with CO_2_, but still showed excellent efficiency. In addition, 4-chloro substituted 2-aminobenzonitrile showed less activity than 5-chloro substituted substrate, which was partly due to the electron withdrawing effect on the basicity. Therefore, the present approach confirmed to be general for the CO_2_ fixation reaction of various structural and electronically diverse 2-aminobenzonitriles and achieved excellent yields of the corresponding quinazolinones as product.

**Table 4:** Synthesis of various quinazolinones^a^

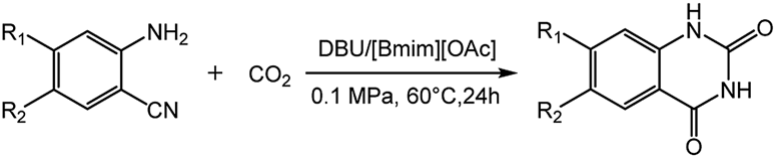			
Entry	Substrate	Product	Yield^b^ (%)
1	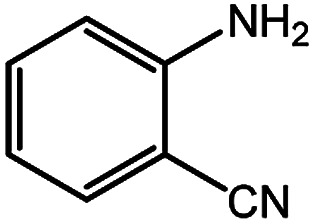	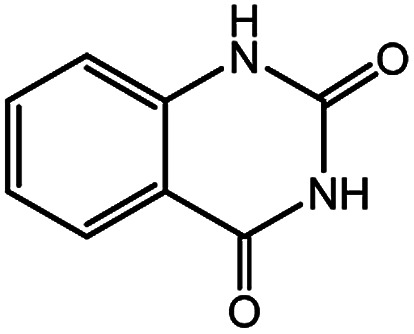	91
2	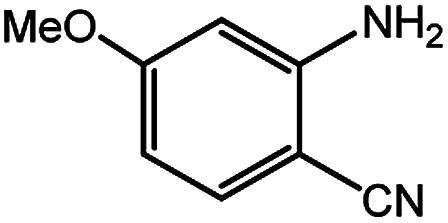	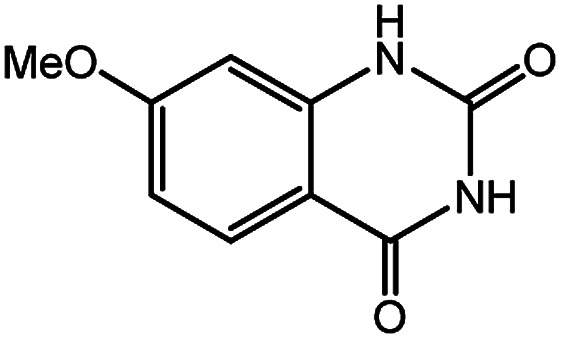	95
3	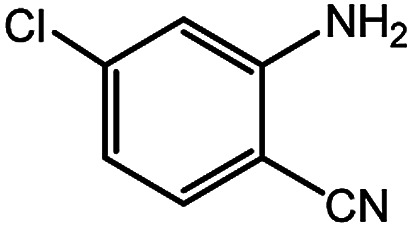	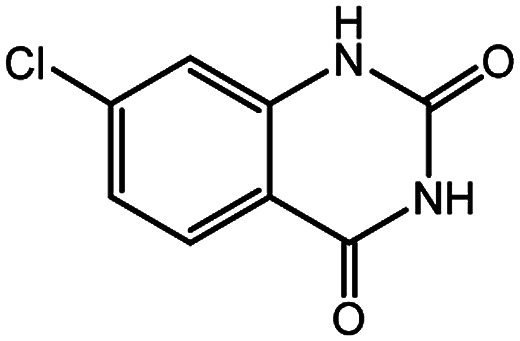	98
4	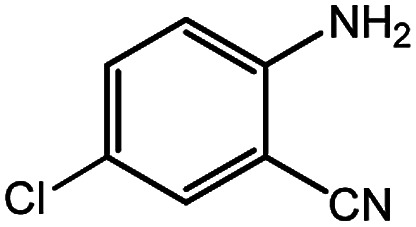	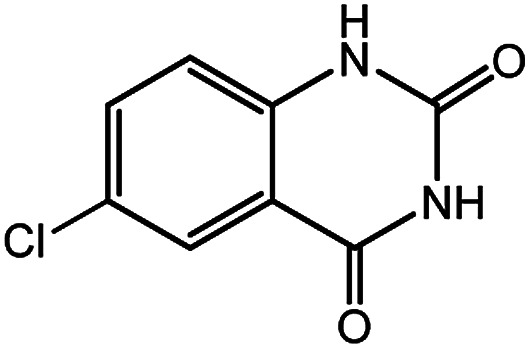	90
5	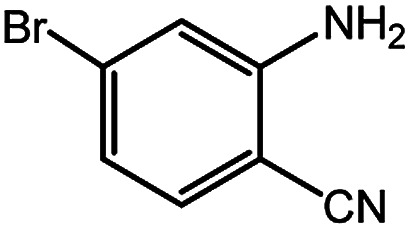	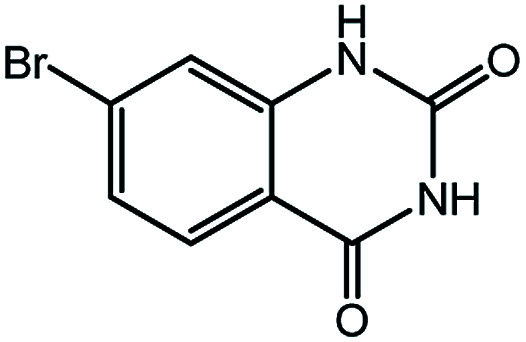	88

a Reaction conditions: reactant 2 mmol, DBU 10 mol%, [Bmim][OAc] 100 mol%, CO_2_ 0.1 MPa, 60 °C, 24 h.

b Isolated yield.

**Fig. 3:**
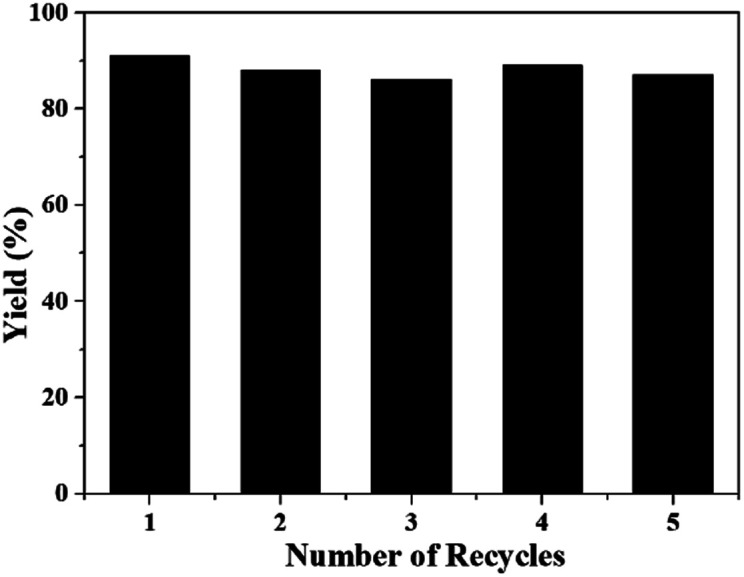
The recyclability of [Bmim][OAc] for the synthesis of quinazolinone. Reaction condition: 2-aminobenzonitrile 2 mmol, DBU 10 mol%, [Bmim][OAc] 100 mol%, CO_2_ 0.1 MPa, 60 °C, 24 h.

**Fig. 4:**
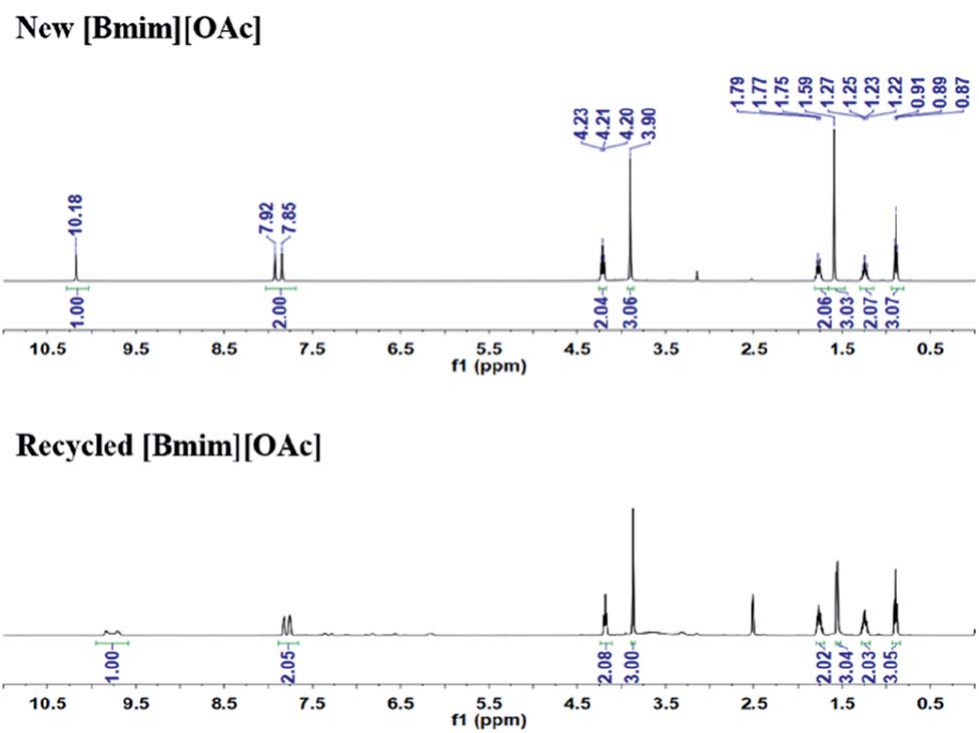
The ^1^H NMR spectra of new and recycled [Bmim][OAc], (DMSO-d_6_, 298 K).

The stability and reusability of [Bmim][OAc] was also tested for the reaction of 2-aminobenzonitriles and CO_2_ under the optimized conditions, and the results were shown in Fig. 3 and 4. Obviously, the yield of quinazolinone almost stayed unchanged as the IL was reused five times (Fig. 3). The ^1^H NMR spectra showed that the structure of the IL was not changed after recycled (Fig. 4), further illustrating that the IL was stable and reusable for the reaction.

**Scheme 2:**
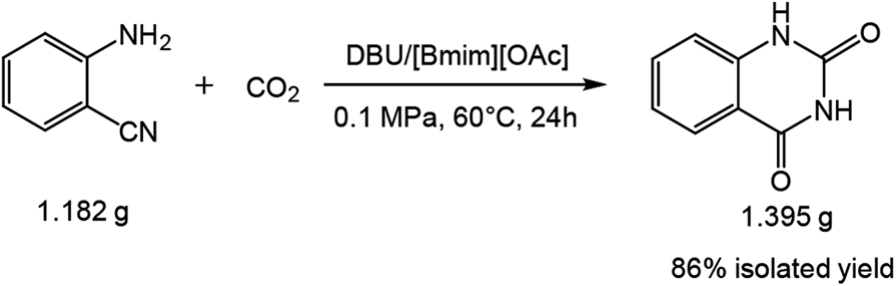
Practical large-scale preparation of quinazolinone. Reaction condition: 2-aminobenzonitrile 10 mmol, DBU 10 mol%, [Bmim][OAc] 100 mol%, CO_2_ 0.1 MPa, 60 °C, 24 h.

In order to demonstrate the synthetic utility of the above approach, the reaction was conducted on a gram scale, and a satisfactory isolated yield of 86% quinazolinone was obtained using 1.182 g substrate. This indicates that the protocol is scalable which may have promising and practical application in the production of quinazolinone (Scheme 2).

**Scheme 3:**
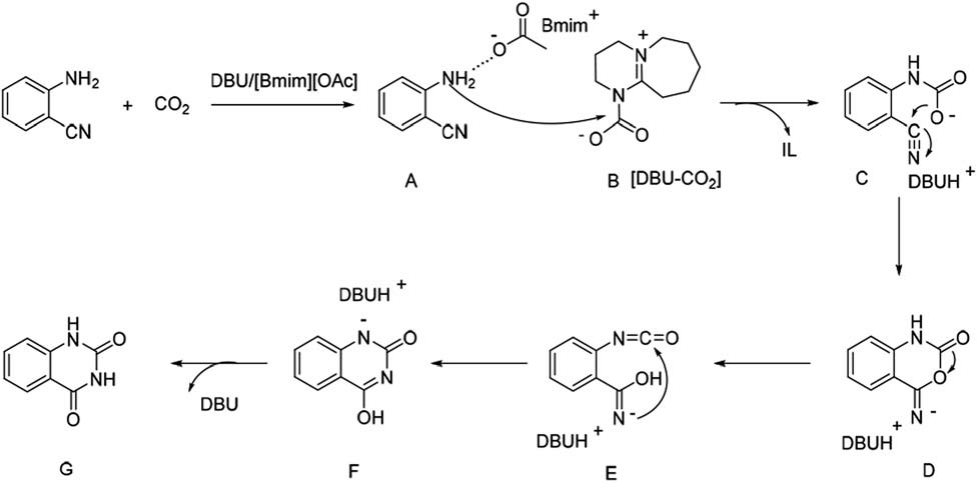
Possible reaction pathway.

It was reported that the NH_2_ group of ortho-substituted aniline could been easily activated *via* hydrogen bond by the [OAc]^−^ anion based ILs,^28^ showing higher nucleophilic reaction capability. The organic bases like DBU or DBN with tertiary nitrogen could react with CO_2_ to form the carbamate species,^14*a*,29^ which resulted in the activation of CO_2_, facilitating the reactions. Hence, based on the experimental results and previous reports, a plausible reaction mechanism for the DBU coupled [Bmim][OAc] catalyzed formation of quinazolinones from 2-aminobenzonitriles with CO_2_ was proposed, as shown in Scheme 3. Firstly, 2-aminobenzonitriles was activated by O atom of the [OAc]^−^ anions of [Bmim][OAc] IL *via* hydrogen bond to form the intermediate A, and CO_2_ was activated by N atom of DBU to form the carbamate intermediate B. The nucleophilic N atom of intermediate A could easily attack the carbon atom of intermediate B to form intermediate C. Then the intermediate D was obtained through the intramolecular nucleophilic cyclization of C, and the isocyanate intermediate E was generated by a ring opening of D. Finally, quinazolinones was produced *via* intramolecular nucleophilic addition and proton transfer.

## Experimental

### Materials

2-Aminobenzonitrile (98%), 2-amino-4-chlorobenzonitrile (98%), 2-amino-5-chlorobenzonitrile (98%), 2-amino-5-bromobenzonitrile (97%), 2-amino-5-methoxybenzonitrile (98%), TMG (tetramethylguanidine, 98%), imidazole (99%), DBU (1,8-diazabicyclo [5.4.0] undec-7-ene, 98%), DBN (1,5-Diazabicyclo [4.3.0] non-5-ene, 99%), Cs_2_CO_3_ (cesium carbonate, 99%), *tert*-butyl methyl ether (99%) were purchased from Saen Chemical Technology Co., Ltd (Shanghai, China). DMF (*N*,*N*-dimethylformamide), DMSO (dimethyl sulfoxide), THF (tetrahydrofuran), acetonitrile were analytic grade and purchased from Shanxi Tongjie Chemical Reagent Co., Ltd (Shanxi, China). NaOH, KOH, Na_2_CO_3_ were analytic grade and purchased from Guangdong Guanghua Sci-Tech Co., Ltd (Guangdong, China). The ILs were supplied by Lanzhou Yulu Fine Chemical Co., Ltd (Gansu, China). All chemicals were used without further purification.

### Typical procedure for the synthesis of quinazolinones

All the reaction were conducted in a single-necked flask of 25 mL equipped with a magnetic stirrer. As an example, the preparation of quinazolinone using 2-aminobenzonitrile and CO_2_ as the raw materials and DBU/[Bmim][OAc] as catalyst was described, and those for other reactions were similar. In a typical reaction procedure, 2-aminobenzonitrile (2 mmol), DBU (10 mol% with respect to substrate), [Bmim][OAc] (100 mol% with respect to substrate) were added into the reactor. After sealing, the air was removed by blowing CO_2_ into the reactor. Then the CO_2_ pressure was kept at 0.1 MPa using a bladder with CO_2_ and the reactor was placed in an oil bath of desired temperature and the reaction mixture was stirred. After a certain time, the reactor was placed into ice water until cool to room temperature. Then 15 mL deionized water was added into the reactor. The mixture was sonicated to rapidly precipitate the product and the aqueous solution of the ILs was removed by suction filtration. The product was washed by water (15 mL) for three times to remove ILs. Then the precipitate was washed by *tert*-butyl methyl ether (15 mL) for three times to remove unreacted substrate from the product. Finally, the quality of the product was determined by an electronic balance (ME204/02) with an accuracy of 0.1 mg after dried at 95 °C for 3 hours and the yield was calculated. In the experiments to test the reusability of the ILs, the catalysts was recovered by evaporating the water and dried at 70 °C for 24 h under vacuum. And the trace amount of DBU remaining in the catalytic system was washed with ether and dried at 40 °C for 1 hour under vacuum to obtain the pure IL. Then the IL was used for the next run.

## Conclusions

In conclusion, a new route for the synthesis of quinazolinones was developed *via* the reaction of 2-aminobenzonitriles with CO_2_ catalysed by DBU coupled ILs catalytic system without other solvents and additives under mild conditions (*e.g.*, 60 °C, 0.1 MPa), and various quinazolinones with different substituent groups were obtained in good to excellent yields. The ILs had high stability and reusability, which can be reused at least five times without considerable decrease in catalytic activity. Moreover, this protocol could been conducted on a gram scale, which may have promising and practical application in the production of quinazolinones.

## Conflicts of interest

There are no conflicts to declare.

## Supplementary Material
